# Fabrication and ultraviolet photoresponse characteristics of ordered SnO*_x _*(*x ≈ *0.87, 1.45, 2) nanopore films

**DOI:** 10.1186/1556-276X-6-615

**Published:** 2011-12-06

**Authors:** Changli Li, Maojun Zheng, Xianghu Wang, Lujun Yao, Li Ma, Wenzhong Shen

**Affiliations:** 1 Laboratory of Condensed Matter Spectroscopy and Opto-Electronic Physics, Department of Physics, Shanghai Jiao Tong University, Shanghai, 200240, People's Republic of China; 2 School of Mechanical Engineering, Shanghai Dianji University, Shanghai 200240, People's Republic of China; 3 School of Chemistry and Chemical Technology, Shanghai Jiao Tong University Shanghai, 200240, People's Republic of China

**Keywords:** highly ordered tin oxide nanopores films, anodized aluminum oxide(aao), ultraviolet(uv) response, oxygen vacancies

## Abstract

Based on the porous anodic aluminum oxide templates, ordered SnO*_x _*nanopore films (approximately 150 nm thickness) with different *x *(*x *≈ 0.87, 1.45, 2) have been successfully fabricated by direct current magnetron sputtering and oxidizing annealing. Due to the high specific surface area, this ordered nanopore films exhibit a great improvement in recovery time compared to thin films for ultraviolet (UV) detection. Especially, the ordered SnO*_x _*nanopore films with lower *x *reveal higher UV light sensitivity and shorter current recovery time, which was explained by the higher concentration of the oxygen vacancies in this SnO*_x _*films. This work presents a potential candidate material for UV light detector.

**PACS**: 81.15.Cd, 81.40.Ef, 81.70.Jb, 85.60.Gz.

## Background

**Figure 1:**
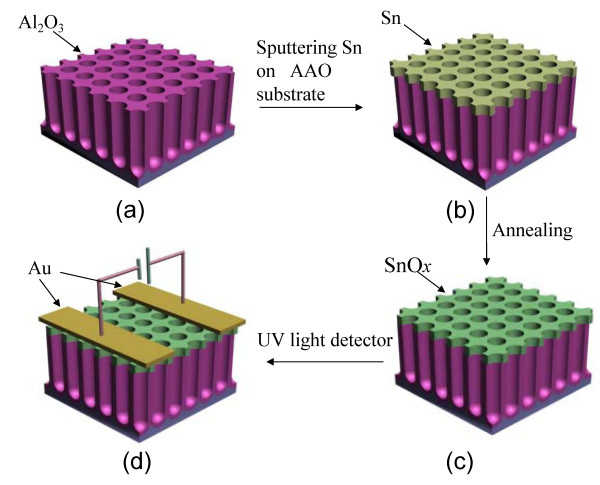
**Schematic diagram of the fabrication process of ordered SnO*_x _*nanopore films**. **(a) **AAO template; **(b) **top view of the ordered tin nanopores array on top of AAO; **(c) **annealing at different temperature; **(d) **ordered SnO*_x _*nanopore-film-based UV photodetector.

Tin oxide is a wide band-gap (3.6 eV) *n*-type semiconductor and exhibits unique electrical and optical properties. It has been used extensively for gas sensors [1-4], solar cells [5], optoelectronic devices [6], catalysts [7], lithium-ion batteries [8], and so forth. In the last few years, intensive attention has been paid to fabricate a variety of SnO_2 _nanostructured materials, such as nanowires [9], nanobelts [10], nanoribbons [11], nanotubes [9,12], nanoparticles [13], and nanowhiskers [14]. However, little attention had been paid to 2D ordered SnO_2 _porous nanomaterials as electronic and chemical devices. 2D ordered porous nanostructures with well-aligned interconnected pores are of great potential applications due to several distinctive properties such as high internal surface areas, high gas sorption and separation capacity, and increased thermal and mechanical stabilities [15]. Herein, we firstly report the fabrication and UV photoconductivity switching properties of highly ordered SnO*_x _*nanopore films. Figure 1 shows the formation process of the highly ordered SnO*_x _*nanopore films. The recovery time of this ordered SnO*_x _*nanopore films for UV detection is much shorter than that of SnO*_x _*thin films and we also found that the films with lower *x *exhibit higher UV sensitivity and faster current recovery. The results indicate that ordered SnO*_x _*nanopore film with low *x *could be as potential candidate material for UV light sensors.

## Methods

The AAO templates were prepared through stable high-field anodization in a H_3_PO_4_-H_2_O-C_2_H_5_OH electrolyte system [16]. Anodization was carried out in a H_3_PO_4_-H_2_O-C_2_H_5_OH electrolyte system (concentration of H_3_PO_4_, 0.25 M) at 195 V. The temperatures of the electrolytes were kept at -10°C to 0°C with a powerful low-constant temperature bath. Sn films were deposited on AAO substrates by direct current (DC) magnetron sputtering using a circular tin target (diameter, 60 mm; purity, 99.99%) at room temperature. The base pressure, deposition pressure, substrate-target distance, sputtering power, and the Ar flux were 1 × 10^-3 ^Pa, 0.85 Pa, 6 cm, 30 W, and 10 sccm, respectively. The sputtering time (*t*) was fixed at 3 min. To obtain the ordered porous SnO*_x _*films and to perform its electrical measurements under UV irradiation, three same samples was annealed at 350°C, 450°C, and 550°C in a quartz tube furnace system for 120 min at a heating rate of 10°C/min, respectively. The quartz tube was evacuated to about 50 Pa before heating and the flow rates of Ar and O_2 _are both fixed at 100 sccm during annealing. Then a 300-nm-thick gold electrodes was evaporated on the surface of SnO*_x _*nanopore films through a shadow mask and copper wires were connected to the electrodes at two contact pads by conducting silver glue. The spacing between the electrodes was 1 mm, and the length of the electrodes was 5 mm. The device structure is depicted in Figure 1d. What's more, SnO*_x _*thin films UV device on the quartz substrate were prepared under the same deposition and post-annealing condition as mentioned above for the purposes of comparison. Electrical measurements of all devices were carried out with a Keithley 2400 source-measure unit under ambient conditions. For UV detection, a xenon lamp was used as the light source and an excitation filter centered at 254 nm and the bias voltage was fixed at 1 V. The structural properties were determined using a D8 DISCOVER X-ray diffractometer (XRD) with Cu Κα radiation. The growth and surface morphologies were observed using a field-emission scanning electron microscope (FE-SEM, Philips Sirion 200, Philips, Holland, Netherlands). The Raman spectra of the SnO*_x _*nanostructures were measured using a Jobin Yvon LabRam HR 800 UV system with a 325 nm He-Cd laser.

## Results and discussions

### Surface topography

**Figure 2:**
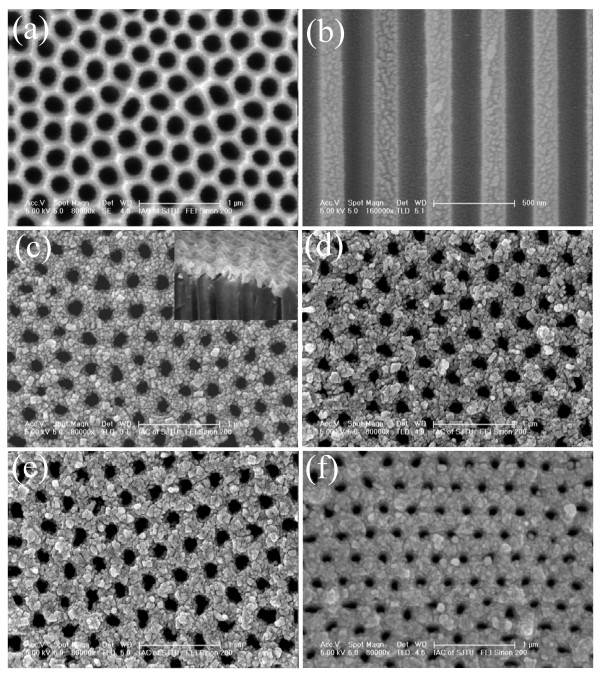
**FE-SEM images of the AAO templates, ordered Sn and SnO*_x _*nanopore films**. **(a) **top view of the alumina template prepared by a two-step anodization method; **(b) **cross-section view of the resultant highly ordered nanochannel alumina template; **(c) **ordered Sn nanopore film prepared at room temperature with P = 30 W and t = 3 min. **(d, f) **the top images of the ordered SnO*_x _*nanopore films prepared at different annealing temperatures (350°C, 450°C, and 550°C, respectively).

Figure 2a, b is the top view and cross-sectional FE-SEM images of a typical AAO template consisting of a hexagonal close-packed arrays formed by the two-step anodization process. The as-grown AAO film has a large pore diameter of approximately 170 nm and interpore spacing of approximately 350 nm, the parallel cylindrical nanochannels can be clearly observed. Figure 2c shows the FE-SEM image of Sn nanopore film on an AAO substrate. The cross-section (he inset of Figure 2c) reveals clearly that the Sn nanopores are on top of the AAO substrates. It is noted that the as-deposited Sn nanopore films consist of small grains and the obtained Sn films just reproduce the substrate geometry. While the pore diameter (approximately 150 nm) of Sn nanopore film is smaller than the pore size of the AAO substrate. Figure 2d, e, f show the surface morphology of the annealed ordered porous Tin films at different temperatures (350°C, 450°C, and 550°C). It can be seen that the grain size of the samples annealed at 350°C was bigger than that of as-deposited Sn films and the neighboring grains seemed to coalescence together as the annealing temperature rises. The surface of the film becomes very smooth and the pore size of the film decreases when annealing temperature was increased to 550°C.

### Composition evolution after annealing at different temperature

**Figure 3:**
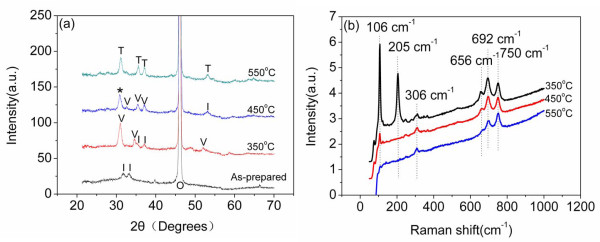
**XRD patterns Raman spectra of SnO*_x _*nanopore films**. **(a) **XRD patterns of the as-prepared sample and SnO*_x _*formed at varying oxidation temperatures of 350°C, 450°C, and 550°C. The symbol (O) indicates the substrate (Al_2_O_3_) reflections. The phases detected in the film are indicated as follows: *I *= Sn; *V *= SnO; asterisk = Sn_2_O_3_; *T *= SnO_2_. **(b) **Evolution of the Raman spectra of SnO*_x _*nanopore films prepared at 350°C, 450°C, and 550°C.

Figure 3a shows the XRD patterns of the Sn nanopore film deposited at room temperature and SnO*_x _*films formed at different annealing temperatures (350°C, 450°C, and 550°C). It can be seen that the as-prepared Sn nanopore film consists only of metallic tin, and SnO is detected with a maximum contribution at 350°C. At the temperature of 450°C, the diffraction profile can be indexed by the reflections of SnO phase and Sn_2_O_3 _phase. This indicates that the SnO and Sn_2_O_3 _are simultaneously present at the annealing temperature of 450°C, and the intergrowth mechanisms may occur at this thermal oxidizing temperature. When the annealing temperature further increases to 550°C, the SnO and Sn_2_O_3 _diffraction peaks disappear, demonstrating that complete SnO_2 _have been formed. This shows that the oxygen content of Tin oxide films prepared by annealing oxidizing is very relative to the annealing temperature. Figure 3b shows the Raman spectra in the Stocks frequency range (50 to 1,000 cm^-1^) for SnO*_x _*films. The Raman spectrum of the sample annealed at 350°C contains strong peaks at approximately 750, 692, 656, 306, 205, and 106 cm^-1^. The strongest peak at 106 and 206 cm^-1^, which is typical of SnO, can be assigned to the B_1g _(113 cm^-1^) and A_1g _(211 cm^-1^) [17]. The bands peaking at 306, 692, and 750 cm^-1 ^can be correspond to SnO_2 _modes E_u_(TO), A_2u_(LO), and E_u_(LO) [18]. In addition to the fundamental Raman scattering peaks of rutile SnO_2_, the other Raman scattering peaks, which are at about 656 cm^-1^, are also observed. The origin of the 656 cm^-1 ^mode, which could not be clearly identified, might indicate other SnO*_x _*stoichiometries. For sample annealed at 450°C, the SnO A_1g _Raman modes disappears and B_1g _Raman modes decreases, indicating the increase of oxygen in structures. Furthermore, the 656 cm^-1 ^mode and SnO B_1g _mode disappear at the annealing temperature of 550°C, which shows the pure SnO_2 _has formed. So the Raman spectra also demonstrate that the oxygen content increasing with the annealing temperature. The EDS analysis during FE-SEM observation reveals that the SnO*_x _*films (prepared at 350°C, 450°C, and 550°C) have an approximate atomic ratio of tin to oxygen of 1:0.87, 1: 1.45, and 1:2, respectively. This is consistent with the results of XRD patterns and Raman spectra.

### Photoresponse of the SnO*_x _*nanopore films under UV irradiation

**Figure 4:**
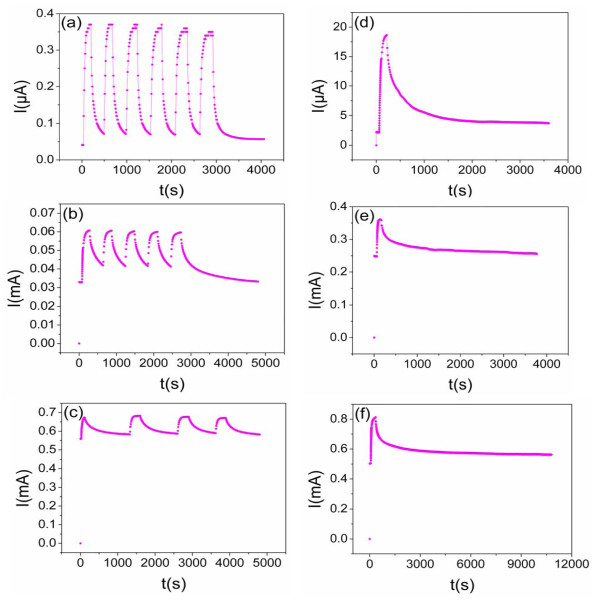
**Time-dependent photoresponse of ordered SnO*_x _*nanopore films and SnO*_x _*thin films**. **(a, c) **Time-dependent photoresponse of ordered SnO*_x _*nanopore films annealed at 350°C, 450°C, and 550°C, respectively. **(d, f) **Time-dependent photoresponse of SnO*_x _*thin films annealed at 350°C, 450°C, and 550°C, respectively. The measurements were carried out in dry air under 1-V bias voltage and approximately 50-μW cm^-2 ^UV illumination.

The room-temperature current-voltage (*I*-*V*) characteristics of the samples all showed a good ohmic behavior and the conductivity of the SnO*_x _*films increase with rising annealing temperature (i.e., increasing film oxygen content) at preparation. To investigate the photoresponse of the ordered SnO*_x _*nanopore films under UV irradiation, the time-dependent measurements of photoresponse were employed to study the rise and decay time upon switching UV light on and off. After keeping the sample in the dark for 60 s under the constant voltage (1 V), we turn on the UV light to reach the maximum of the photocurrent and then turn off the light to observe its recovery characteristics. The reproducibility of the sample was tested by repeatedly switching UV light on and off for the same time intervals. At the last cycle of the measurement, the photocurrent naturally returns to original value. Figure 4a, c shows the time evolution of current under UV lamp irradiation of a power of *I *= 50 μW cm^-2 ^at RT in air. The time-dependent photoresponse of sample prepared at 350°C reveals a current increase steeply upon switching on UV light of more than ten times of magnitude (Figure 4a). The response time, defined as the time needed to reach 90% of the maximum photocurrent, was therefore about 52 s, and recovery time, defined as the time taken for the photocurrent to come within 10% of the initial value, about 270 s. Figure 4b shows the time-dependent photoresponse of the sample prepared at 450°C. The response time and the recovery time were approximately 70 s and approximately 1,090 s, the current increase is about two times of magnitude in this case. For sample prepared at 550°C, a response time of approximately 62 s and a recovery time of approximately 3,350 s were obtained and the current increase is no more than one time of magnitude (Figure 4c). For SnO*_x _*thin films, the response time and recovery time of the samples prepared at 350°C, 450°C, and 550°C are approximately 80 and 2,850 s, approximately 32 and 3,010 s, approximately 100 and > 10,450 s, respectively (Figure 4d, e, f). These results demonstrated that the ordered SnO*_x _*nanopore film prepared at lower temperature possess higher UV light sensitivity and shorter current recovery time. What's more, the recovery time of ordered nanopore films is much shorter than that of thin films and reveal a good reversible switching characteristics with on/off UV exposure. Compared to SnO_2 _nanowire-based UV detectors, the response and recovery performance of our UV detector (prepared at 350°C) was comparable to the SnO_2 _nanowire array-based UV detector [19] and was still inferior to single nanowire UV detectors (the response time is less than 0.1 s) [20]. So there is still much work to do to further improve the performance of ordered SnO*_x _*nanopore-film-based UV detectors in order to meet the practical application.

The decreased recovery time of the ordered SnO*_x _*nanopore films compared to the thin films can be attributed to the increased surface areas. It is known that the oxygen molecules are absorbed onto SnO*_x _*surface by capturing free electrons from the *n*-type SnO*_x _*[O_2_(g) + e^- ^→ O_2_^-^(ad)], which decrease the carrier density in the films and hence the porous films show a higher resitance. Upon UV illumination, electron-hole pairs are generated. The holes migrate to the surface along the potential slope produced by the band bending and recombine with the negatively charged adsorbed oxygen ions [h^+^+O_2_^- ^→ O_2_(g)], resulting in an enhancement of photocurrent. When the illumination is turned off, the films with higher surface area make O_2 _readsorbed on the surface easier, which lead to a shorter recovery time.

For the sample with a lower annealing, temperature shows a shorter recovery time, which could be attributed to below two main processes. First, it is known that oxygen vacancies in SnO*_x _*act as electron donors and the number of oxygen vacancies is expected to increase in lower annealing temperature under certain oxygen flows and annealing time (confirmed by the results of XRD pattern and Raman spectra above), higher concentration of the oxygen vacancies will give higher probability of the adsorption of oxygen molecules onto the surface of SnO*_x _*films, leading to the fast decreasing of the photocurrent. Second, the increase in the oxygen vacancies is expected to decrease the bending of the semiconductor near the surface [21]. Electrons and holes recombine more easily with less bended band, inducing a shorter carrier lifetime. So the photocurrent decay after switching off UV is faster for the sample at lower annealing temperature.

## Conclusions

In conclusion, we firstly report an effective method for the fabrication of ordered SnO*_x _*nanopore films. Annealing temperature is the key factor to control Sn/O ratio. Reversible photoconductive switching characteristics of the films were exhibited by switching UV light on/off, which is ascribed to the oxygen desorption/reabsorption on the surface of SnO*_x _*film. It is noted that the ordered SnO*_x _*nanopore films with lower *x *value possess more excellent ability to detect weak UV light, which could be attributed to the higher concentration of the oxygen vacancies in this SnO*_x _*films. Especially, this ordered nanopore films exhibit shorter recovery time compared to the thin films, which can be attributed to the increased surface areas. This study presents a new approach for fabricating UV light sensors based on Tin oxide films.

## Abbreviations

AAO: anodized aluminum oxide; UV: ultraviolet; 2D: two-dimensional; DC: direct current; XRD: X-ray diffraction; FE-SEM: field-emission scanning electron microscope; EDS: energy disperse spectroscopy; RT: room temperature.

## Competing interests

The authors declare that they have no competing interests.

## Authors' contributions

CLL participated in the design of the study, carried out the total experiments, performed the statistical analysis, as well as drafted the manuscript. MJZ participated in the design of the study, provided the theoretical and experimental guidance, performed the statistic analysis, and revised the manuscript. XHW helped to operate the Magnetron Sputtering System. LM participated in the design of experimental section and offered her the help in experiments. LJY provided helpful suggestion in the analysis of experimental data. WZS gave his help in the setting up of experimental apparatus. All authors read and approved the final manuscript.
